# Supplementary material for: Patterns of Intron Gain and Loss in Fungi
Source: PLoS Biol. 2004 Nov 30;2(12):e422. doi: 10.1371/journal.pbio.0020422 (PMC532390; doi:10.1371/journal.pbio.0020422)
Supplement: Table S1 — Also available at http://genes.mit.edu/NielsenEtAl/. (4.3 MB ZIP). [file pbio.0020422.st001.zip › NielsenEtAl/html/1319.html]

AN1182.1.NCU04054.1.MG00604.2.FG09530.1


```
 CLUSTAL W (1.82) Multiple Sequence Alignments - Introns Inserted


Sequence 1: NCU04054.1	447 aa
Sequence 2: MG00604.2	447 aa
Sequence 3: FG09530.1	446 aa
Sequence 4: AN1182.1	447 aa
Alignment Length: 447 aa
Number Identitical Residues: 410 aa
Alignment Score (without introns) 16155


MG00604.2 	MREI0VHLQTGQC0GNQIGAAFW2Q~TISSEHGLDSNGV2YNGTSELQLERMSVYFNE0A
NCU04054.1	MREI0VHLQTGQC0GNQIGAAFW~Y0TISGEHGLDASGV2YNGTSELQLERMNVYFNE0A
FG09530.1 	MREI0VHLQTGQC0GNQIGAAFW~Q~TISGEHGLDSNGV~YNGTSELQLERMSVYFNE0A
AN1182.1  	MREI0VHLQTGQC0GNQIGAAFW2Q~TISGEHGLDGSGV2YNGTSDLQLERMNVYFNE0A
          	**** ******** *********   ***.*****..** *****:******.***** *

MG00604.2 	SGNKHVPRAVLVDLEPGTMDAVRAGPFGQLFRPDNFVFGQSGAGNNWAKGHYTEGAELVD
NCU04054.1	SGNKYVPRAVLVDLEPGTMDAVRAGPFGQLFRPDNFVFGQSGAGNNWAKGHYTEGAELVD
FG09530.1 	SGNKYVPRAVLVDLEPGTMDAVRAGPFGQLFRPDNFVFGQSGAGNNWAKGHYTEGAELVD
AN1182.1  	SGNKYVPRAVLVDLEPGTMDAVRAGPFGELFRPDNFVFGQSGAGNNWAKGHYTEGAELVD
          	****:***********************:*******************************

MG00604.2 	QVLDVVRREAEGCDCLQGFQITHSLGGGTGAGMGTLLISKIREEFPDRMMATFSVVPSPK
NCU04054.1	QVLDVVRREAEGCDCLQGFQITHSLGGGTGAGMGTLLISKIREEFPDRMMATFSVVPSPK
FG09530.1 	QVLDVVRREAEGCDCLQGFQITHSLGGGTGAGMGTLLISKIREEFPDRMMATFSVVPSPK
AN1182.1  	NVVDVVRREAEGCDCLQGFQITHSLGGGTGAGMGTLLISKIREEFPDRMMATFSVVPSPK
          	:*:*********************************************************

MG00604.2 	VSDTVVEPYNATLSVHQLVENSDETFCIDNE~ALYDICMRTLKLSNPSYGDLNYLVSAVM
NCU04054.1	VSDTVVEPYNATLSVHQLVENSDETFCIDNE~ALYDICMRTLKLSNPSYGDLNHLVSAVM
FG09530.1 	VSDTVVEPYNATLSVHQLVENSDETFCIDNE~ALYDICMRTLKLSNPSYGDLNYLVSAVM
AN1182.1  	VSDTVVEPYNATLSVHQLVEHSDETFCIDNE0ALYDICMRTLKLSNPSYGDLNHLVSAVM
          	********************:********** *********************:******

MG00604.2 	SGVTTCLRFPGQLNSDLRKLAVNMVPFPRLHFFMVGFAPLTSRGAHSFRAVTVPELTQQM
NCU04054.1	SGVTVSLRFPGQLNSDLRKLAVNMVPFPRLHFFMVGFAPLTSRGAHHFRAVSVPELTQQM
FG09530.1 	SGVTTCLRFPGQLNSDLRKLAVNMVPFPRLHFFMVGFAPLTSRGAHSFRAVSVPELTQQM
AN1182.1  	SGVTTCLRFPGQLNSDLRKLAVNMVPFPRLHFFMVGFAPLTSRGAYSFRAVSVPELTQQM
          	****..***************************************: ****:********

MG00604.2 	FDPKNMMAASDFRNGRYLTCSAIF2RGKVSMKEVEDQMRNVQNKNSSYFVEWIPNNIQTA
NCU04054.1	FDPKNMMAASDFRNGRYLTCSAIF2RGKVSMKEVEDQMRNVQNKNSSYFVEWIPNNVQTA
FG09530.1 	FDPKNMMAASDFRNGRYLTCSAIF~RGRVAMKEVEDQMRNVQSKNSSYFVEWIPNNIQTA
AN1182.1  	FDPKNMMAASDFRNGRYLTCSAIF2RGKVSMKEVEDQMRNIQSKNQSYFVEWIPNNIQTA
          	************************ **:*:**********:*.**.**********:***

MG00604.2 	LCSIPPRGLKMSSTFIGNSTAIQELFKRVGEQFTAMFRRKAFLHWYTGEGMDEMEFTEAE
NCU04054.1	LCSIPPRGLKMSSTFVGNSTAIQELFKRIGEQFTAMFRRKAFLHWYTGEGMDEMEFTEAE
FG09530.1 	LCAIPPRGLTMSSTFIGNSTSIQELFKRVGEQFTAMFRRKAFLHWYTGEGMDEMEFTEAE
AN1182.1  	LCSIPPRGLKMSSTFIGNSTSIQELFKRVGDQFTAMFRRKAFLHWYTGEGMDEMEFTEAE
          	**:******.*****:****:*******:*:*****************************

MG00604.2 	SNMNDLVSEYQQYQDAGVDEEEEEY~EEEAPLEGEE
NCU04054.1	SNMNDLVSEYQQYQDAGVDEEEEEY~EEEAPLEGEE
FG09530.1 	SNMNDLVSEYQQYQDAGIDEEEEEY~EEELP-EGEE
AN1182.1  	SNMNDLVSEYQQYQDASISEGEEEY2AEEEIMEGEE
          	****************.:.* ****  **   ****
```
